# Supplementary figures and images for: Integrated Metabolomic and Transcriptomic Analysis of Puerarin Biosynthesis in Pueraria montana var. thomsonii at Different Growth Stages
Source: Genes (Basel). 2023 Dec 18;14(12):2230. doi: 10.3390/genes14122230 (PMC10742406; doi:10.3390/genes14122230)

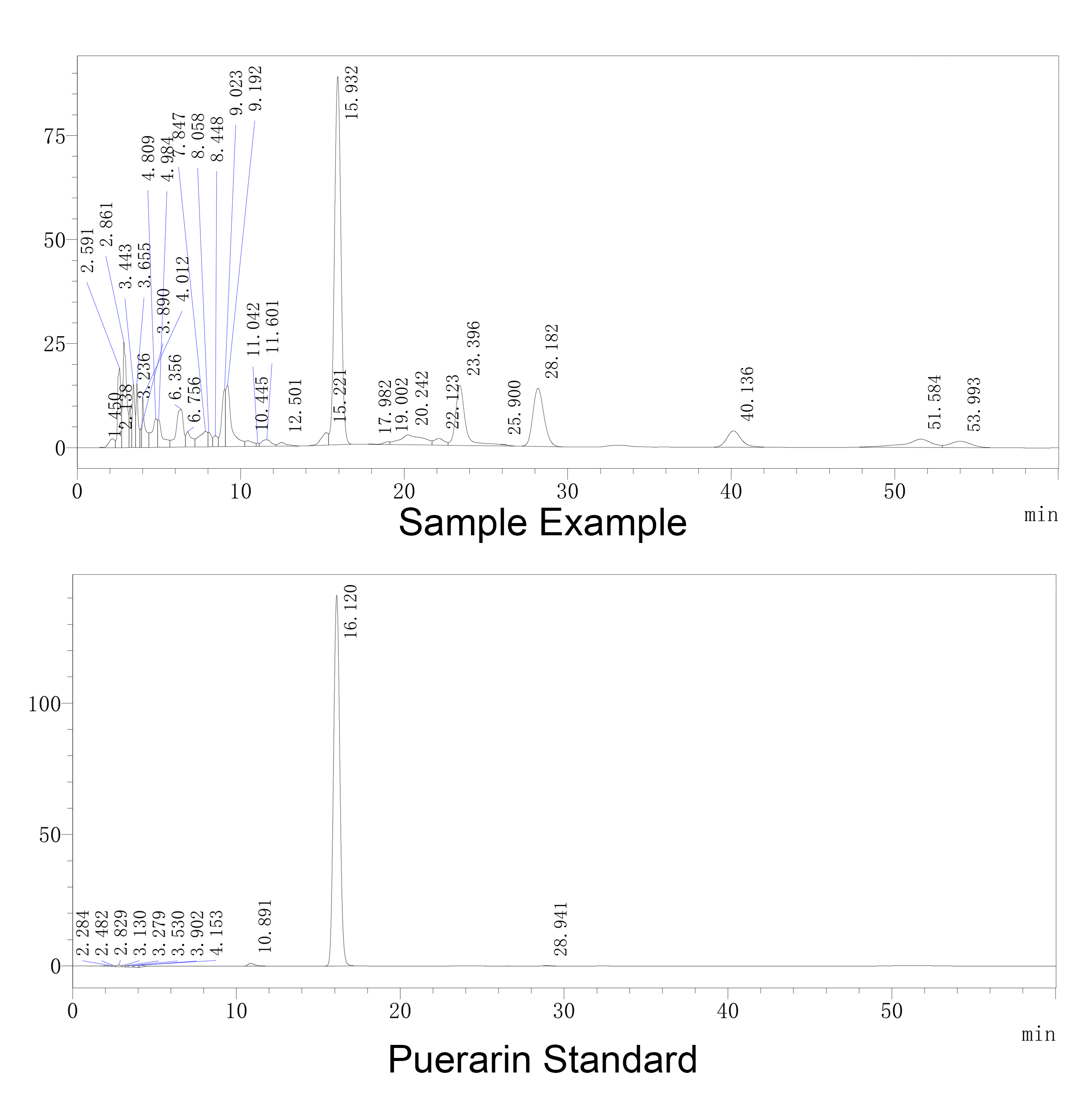

Supplement: Supplementary file 1 [file genes-14-02230-s001.zip › FigureS1.tif]

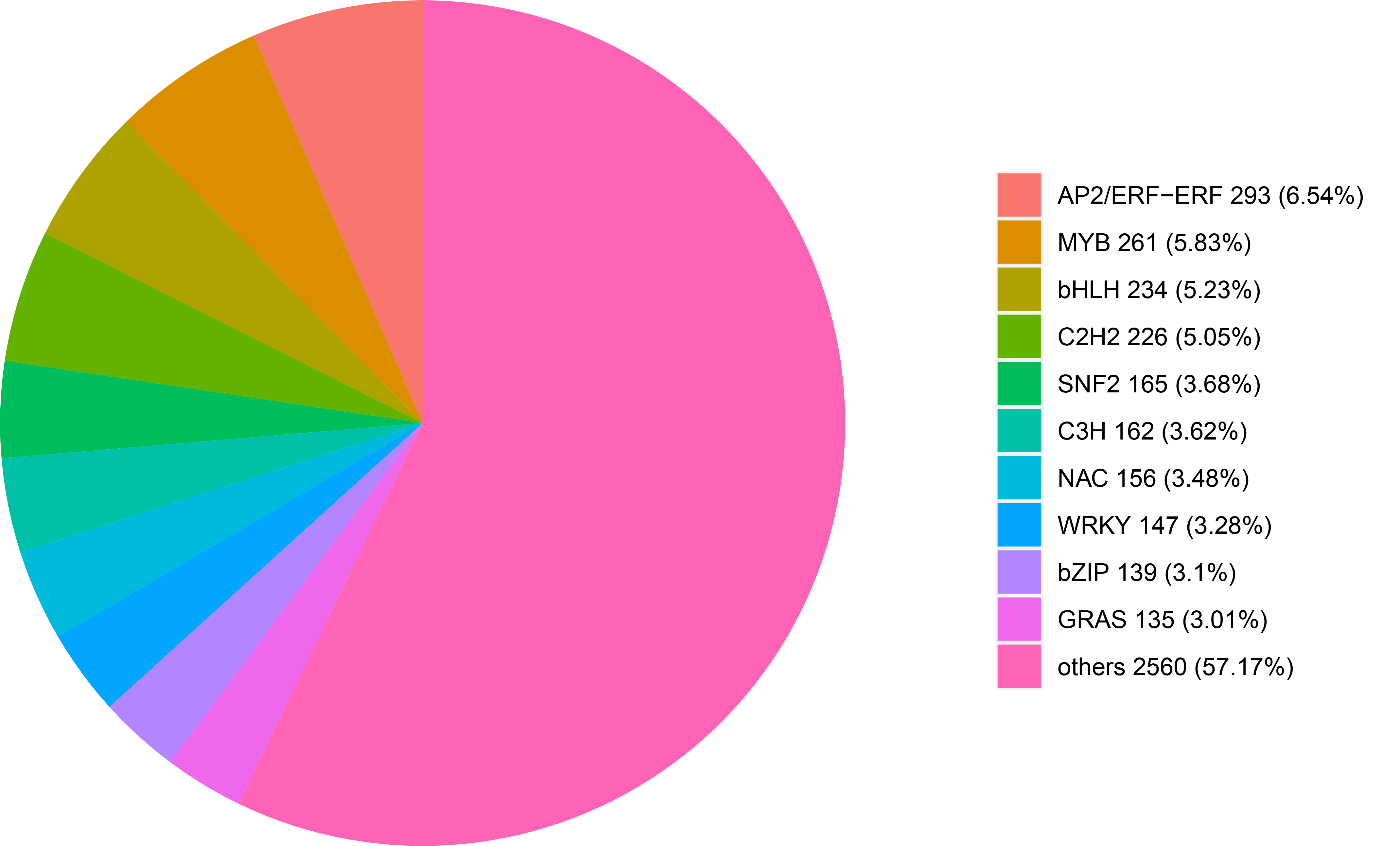

Supplement: Supplementary file 1 [file genes-14-02230-s001.zip › FigureS2.tif]
